# Extensive Variation in Drought-Induced Gene Expression Changes Between Loblolly Pine Genotypes

**DOI:** 10.3389/fgene.2021.661440

**Published:** 2021-05-31

**Authors:** Jingjia Li, Jason B. West, Alexander Hart, Jill L. Wegrzyn, Matthew A. Smith, Jean-Christophe Domec, Carol A. Loopstra, Claudio Casola

**Affiliations:** ^1^Department of Ecology and Conservation Biology, Texas A&M University, College Station, TX, United States; ^2^Department of Ecology and Evolutionary Biology, University of Connecticut, Storrs, CT, United States; ^3^Department of Biological Sciences, Florida International University, Miami, FL, United States; ^4^Bordeaux Sciences Agro, UMR 1391 INRA ISPA, Gradignan, France; ^5^Nicholas School of the Environment, Duke University, Durham, NC, United States

**Keywords:** drought tolerance, *Pinus taeda*, RNA-seq, GxE, differential gene expression

## Abstract

Drought response is coordinated through expression changes in a large suite of genes. Interspecific variation in this response is common and associated with drought-tolerant and -sensitive genotypes. The extent to which different genetic networks orchestrate the adjustments to water deficit in tolerant and sensitive genotypes has not been fully elucidated, particularly in non-model or woody plants. Differential expression analysis via RNA-seq was evaluated in root tissue exposed to simulated drought conditions in two loblolly pine (*Pinus taeda* L.) clones with contrasting tolerance to drought. Loblolly pine is the prevalent conifer in southeastern U.S. and a major commercial forestry species worldwide. Significant changes in gene expression levels were found in more than 4,000 transcripts [drought-related transcripts (DRTs)]. Genotype by environment (GxE) interactions were prevalent, suggesting that different cohorts of genes are influenced by drought conditions in the tolerant vs. sensitive genotypes. Functional annotation categories and metabolic pathways associated with DRTs showed higher levels of overlap between clones, with the notable exception of GO categories in upregulated DRTs. Conversely, both differentially expressed transcription factors (TFs) and TF families were largely different between clones. Our results indicate that the response of a drought-tolerant loblolly pine genotype vs. a sensitive genotype to water limitation is remarkably different on a gene-by-gene level, although it involves similar genetic networks. Upregulated transcripts under drought conditions represent the most diverging component between genotypes, which might depend on the activation and repression of substantially different groups of TFs.

## Introduction

Low water availability affects productivity and growth in both natural forests and tree plantations and is expected to become a primary limiting factor in areas subject to local climate shifts (Karl et al., 2009). The combination of decreasing precipitation and increasing temperatures and atmospheric water demand will likely exert a strong selective pressure on natural tree populations. Plant response to drought occurs across numerous traits and can be observed at several organizational scales (e.g., cellular, tissue, whole plant). Variation in drought tolerance between populations is the result of the adaptation to local environmental conditions and several fundamental physiological or morphological trade-offs, particularly in species with broad ranges (Sánchez-Salguero et al., 2018), wherein genotypes with high and low tolerance to water limitation can evolve in response to the local climate (Blackman et al., 2017). Thus, investigating the genetic basis of drought tolerance in species with populations adapted to a variety of water availability conditions is essential to help understand how plants respond to this abiotic stress. Additionally, species with large populations and locally adapted varieties may benefit from the migration of drought-tolerant genotypes toward areas that will become increasingly more prone to water deficit due to climate change (Aitken et al., 2008).

Loblolly pine (*Pinus taeda* L.) represents the most commonly planted tree across the southeastern United States (Smith et al., 2009), where rainfall is projected to become more variable, resulting in prolonged drought periods (Mbow et al., 2017). Local adaptation in loblolly pine has been documented by a number of studies on several phenotypic traits (Eckert et al., 2010; Quesada et al., 2010; Cumbie et al., 2011; Palle et al., 2011), including tolerance to aridity (Gonzalez-Martinez et al., 2008; Eckert et al., 2010) and differences among genotypes in response to soil drying (Aspinwall et al., 2011; Wilson, 2014). For example, Eckert et al. identified five loci associated with levels of aridity in *P. taeda* using 3059 SNPs (Eckert et al., 2010). Exome- and genome-wide polymorphisms are now available for loblolly pine (Lu et al., 2016; De La Torre et al., 2018, 2019), enabling the identification of a large number of variants associated with traits and/or climate variables. Genotype–phenotype association studies based on these data have revealed a few SNPs and SNP–SNP epistatic interactions associated with Δ^13^C, a proxy for water use efficiency (Lu et al., 2017). Additionally, 611 SNPs were found to be associated with 56 climate and geographic variables, including several hundred that associated with temperature and precipitation variables (Lu et al., 2019). The combined analysis of exome polymorphisms, gene expression, and metabolomic studies has led to the identification of 661 SNPs associated with drought-related genes (Lu et al., 2018). Using over 87,000 variants obtained from whole genome resequencing, De La Torre and collaborators also reported that water availability represents the primary climate variable associated with local adaptation in loblolly pine (De La Torre et al., 2018, 2019).

Genes associated with drought tolerance in plants have also been identified by assessing variation in gene expression in controlled experiments, including water-deficit stress treatments of genotypes with varying tolerance to aridity. This strategy has revealed that the expression level of thousands of genes from a multitude of genetic networks are significantly affected in response to prolonged low water availability (Cohen et al., 2010; Des Marais et al., 2012; Zhang et al., 2014; Shin et al., 2015; Miao et al., 2017; Xu et al., 2018). In loblolly pine, studies of gene expression changes induced by drought stress have been conducted for more than two decades. Early investigations in seedlings exposed to drought stress have shown expression changes in genes encoding S-adenosylmethionine synthetase, transcription factors (TFs) belonging to the ABA pathway, glycoproteins, and a glycine-rich protein associated to the cell wall (Chang et al., 1996). Further studies have pointed to changes in the activity of genes encoding stress-response proteins, including heat shock proteins, dehydrins, and other late embryogenic-abundant (LEA) proteins, as well as enzymes involved in several metabolic pathways (Watkinson et al., 2003; Lorenz et al., 2006). In one of the most comprehensive analyses of gene expression in drought-stressed loblolly pine, Lorenz and collaborators identified multiple genetic networks involved in drought response, including 9-*cis*-epoxycarotenoid dioxygenase, zeatin *O*-glucosyltransferase, and ABA-responsive proteins (Lorenz et al., 2011). Analogous investigations in other conifers have largely mirrored these findings (Moran et al., 2017). Importantly, the expression level of these genes was comparable in control and drought seedlings following re-watering of water stressed plants (Watkinson et al., 2003; Lorenz et al., 2006; Lorenz et al., 2011).

Overall, genes with similar functions have been found to be over- or under-expressed in both flowering plants and gymnosperms grown in water-deficit conditions. These genes are involved in an array of cellular processes activated by drought stress, including protection from oxidative, heat and osmotic stress, changes in metabolic functions, transcription regulation, and release of hormones and other signaling molecules (Moran et al., 2017). Similar results have been reported in microarray or transcriptomic studies of other drought-stressed conifers, including *Pinus pinaster* and *Pinus pinea* (Perdiguero et al., 2013), *Pinus halepensis* (Fox et al., 2018), *Abies alba* (Behringer et al., 2015), *Pseudotsuga menziesii* (Muller et al., 2012), and *Cunninghamia lanceolata* (Hu et al., 2015).

To provide a comprehensive description of the genes involved in drought response in loblolly pine, we performed a transcriptomic analysis of control and drought-stressed root systems from two loblolly pine clones with different physiological responses to drought. We identified more than 4000 transcripts with significant changes in expression level in seedlings grown under drought conditions in either clone. Few of these drought-related transcripts (DRTs) were shared between the clones, indicating extensive genotype by environment (GxE) interactions between these drought-tolerant and -sensitive loblolly pine genotypes. Although GxE interactions were less prevalent at the level of functional gene annotations (GO terms) and metabolic pathways, they were common among TFs and TF families encoded by DRTs. These findings revealed an unexpected divergence in the genetic networks involved in the response to water deficit between loblolly pine genotypes.

## Materials and Methods

### Plant Materials and Experimental Design

The loblolly pine genotypes were provided by ArborGen Inc. A total of 140 ramets (20 for each variety) were planted on September 25, 2014, in a greenhouse operated by the Department of Ecosystem Science and Management at Texas A&M University in College Station, TX. After 4 weeks of growth in well-watered conditions, ramets of each variety were randomly assigned to five blocks (replicates) for each of two treatments, well-watered (control) and low-watered (drought-simulated). The drought-simulated ramets received a watering frequency of about 17% of the control ramets (watered once for every six times the control ramets were watered). These simulated droughts were applied in two periods: from December 2014 to March 2015 and from mid-April 2015 to the end of May 2015. All ramets were grown in sand with fertilizer added approximately every 2 months [760 mg/L Peter’s 20-20-20, 6 mg/L Peter’s STEM micronutrient, 170 mg/L Ferriplus (6% Fe), and 420 mg/L Epsom salts (9.6% Mg and 14.5% S)], with automatic watering adjusted based on soil moisture and pre-dawn water potential measurements. Ramets from the three genotypes 2, 5, and 6 were selected for further growth and sampling based on gas exchange preliminary data taken in December 2014 showing differences between varieties in stomatal conductance and photosynthetic rate. After 6 months, genotypes 2 and 5 showed the highest difference in several physiological traits and were selected for phenotype and transcriptome (RNA sequencing) analyses (Supplementary Table 1 and Supplementary Figures 1, 2). Six ramets from each of the three genotypes (three ramets per treatment) were harvested on the morning of May 29, 2015. Harvested tissues to be used for transcriptome analyses were immediately stored in a −80°C freezer.

### Physiological Measurement and Treatment Comparison

Leaf-level physiological measurements conducted at or immediately before harvest included pre-dawn water potential (Ψ_*PD*_ and Ψ_*MD*_; Model 1000, PMS Instrument Company, Albany, OR, United States), leaf N isotopic composition (δ^15^N; ‰), and concentration (%N) that were taken as a proxy for plant nitrogen assimilation and leaf photosynthetic activity (Evans, 2001), and bulk leaf C isotopic composition (δ^13^C; ‰) was taken as an indicator of intrinsic water-use efficiency (Lin et al., 2019). Leaves were ground to powder using a Ball Mill (MM 400; Retsch GmbH, Haan, Germany), placed in tin capsules, and weighed prior to isotope analysis (EA-IRMS; Costech Analytical Technologies Inc., Valencia, CA, United States). Calibration standards were as follows: USGS 40, d^13^C = −26.39‰, d^15^N = −4.52‰ and USGS 41, ^13^C = 36.55‰, d^15^N = 47.55‰. All sample processing and stable isotope analysis was performed at the Stable Isotope for Biosphere Science (SIBS) Laboratory^1^ at Texas A&M University. Additionally, the following measures of stem hydraulics (see Wilson, 2014; Domec et al., 2015 for more details) were performed on six samples per clone and per treatment: maximum specific hydraulic conductivity (*K*_*s*_; kg m^–1^ s^–1^ MPa^–1^), water potential at which 50% of stem *K*_*s*_ is lost (P_50_; MPa), and drought-induced cavitation sensitivity (change in percent loss in stem *K*_*s*_ per unit water potential; % MPa^–1^). Dry plant biomass and stem wood density (dry mass over fresh volume; g cm^–3^) at harvest was also quantified. Dry mass measured following drying to constant weight in oven at 60°C.

### RNA Extraction and cDNA Sequencing

Total RNA was extracted from whole needles and part of the root system (∼100 mg each) for each harvested ramet. After grinding each sample in liquid nitrogen, total RNA was isolated using the Sigma Plant RNA/DNA Purification Kit. The Qiagen DNase I kit was used to digest gDNA. Total RNA integrity was assessed by electrophoresis on 1% agarose gels under denaturing conditions (Supplementary Figure 3). RNA of each sample was further analyzed using a NanoDrop 1000 instrument. RNA samples with *A*_260_/*A*_280_ between 1.8 and 2.1 and RQN between 5.2 and 10.0 were used for RNA-sequencing (RNA-seq) experiments. Quality control, library preparation, sequencing, and preliminary data filtering were performed by the Texas A&M AgriLife Genomics and Bioinformatics Services. In total, 24 RNA samples were processed to build libraries for RNA-seq analysis using the Illumina TruSeq RNA Sample Preparation Kit, as per manufacturer instructions (Supplementary Table 2). All libraries were quality checked and sequenced on two lanes of Illumina HiSeq-2500 platform using a 2 × 125-bp paired-end strategy. One needle library contained mostly bacterial DNA and was thus removed from downstream analyses. Sequencing of the 23 remaining samples generated 568.2 million raw reads (∼120 Gb) reduced to 514.6 million reads after pre-filtering (see below). The average reads number was 24,992,695 and 19,518,450 for each root and needle library, respectively.

### Reads Data Filtering

More than 95% of de-multiplexed reads passed the instrument-level pre-filtering and were further processed. The pre-filtered reads were checked using FastQC v0.11.5 (Leggett et al., 2013). Filtering was applied to the raw data to generate clean reads with the following approach. First, the program SortMeRNA v2.1 (Kopylova et al., 2012) was used to identify and remove reads corresponding to rRNA genes. On average, 4.24% of reads were removed from each library in this step. Second, adapters were cut from the reads allowing maximally two mismatches under the quality score threshold of 30 using Trimmomatic version 0.35 (Bolger et al., 2014). Reads were scanned with a 4-base wide sliding window and cut when the average quality per base drops below 14, and reads with less than 50 bases long after the trimming steps were dropped. Finally, we implemented a stringent filtering process after mapping reads onto the genome assembly v1.01, in order to account for the high level of sequence redundancy in the large loblolly pine genome. Cleaned reads from the previous two steps were aligned to the loblolly pine genome v1.01 using HiSAT2 v2.0.5 (Kim et al., 2015), applying default parameters except min-intronlen and max-intronlen set to 30 and 10,000,000, respectively. Subsequently, we removed reads that do not map concordantly on a single locus or have >3 mismatches by retaining only reads with the following parameters in the SAM output: NH:i:1, YT:Z:CP, and XM:i:0-3. This step allowed reducing the mapping of reads to incorrect loci.

### Transcriptome Assemblies

A genome-guided reference transcriptome was built from all the clean reads stringently mapped onto the assembly v1.01 (see section above) using StringTie v1.3.1 (Pertea et al., 2015), which assembles and quantifies the transcripts including novel splice variants in each library. A combined assembly was then generated using the Stringtie merge function to construct one set of transcripts, which was consistent across all 46 samples with better read coverage. The final transcriptome assembly consisted of 99,756 transcripts. Candidate coding regions were retrieved from 54,826 transcripts using TransDecoder v3.0.1^2^ based on merged transcript sequences (Supplementary Table 3). The transcript abundances for each library were re-computed by StringTie based on the newly constructed candidate coding transcriptomic structure. The filtered high-quality reads were assembled and merged by Stringtie to get a total number of 54,826 transcripts with an N50 length of 1440 bp. The re-estimation from the assembly results of each library against the merged transcriptomic data was carried out, resulting in transcripts expression value count matrix.

### Genetic Distance

SNPs between each library and the loblolly pine assembly v1.01 reference sequence were detected using the programs Opossum v0.1 and Platypus v0.8.1 (Oikkonen and Lise, 2017). Opossum was used to pre-process the assembled data for each library, whereas variant-detection calling was carried out with Platypus using reads realignment to the genome assembly to achieve both high sensitivity and high specificity. Candidate variants were filtered based on PASS and Quality of 100 or above, and then the ones supported by a minimum of 10 reads coverage were kept by Platypus. Genetic distances were calculated as the number of SNPs divided by the total number of aligned nucleotides between each library and the genome assembly (Supplementary Table 4).

### Quantitative RT-qPCR

Twenty-two transcripts with varying degrees of differential expression between control and drought-stressed ramets were selected for RT-qPCR experiments (Supplementary Table 5). The primers were designed to be 21–27 bp long using the Primer3 web version 4.1.0 (Untergasser et al., 2012) with an *e*-value < 2e–04 and score > 41. Two transcripts encoding for the elongation factor 1-alpha and the ubiquitin-activating enzyme E1were used as internal controls to normalize the expression values based on their limited variation in expression (| log_2_ fold change| < 0.15) and consistently high expression level across tissues and treatments (Supplementary Table 5). The relative quantitative method (ΔΔCT) was used to calculate the fold change in the expression levels of target genes (Livak and Schmittgen, 2001). RT-qPCRs were performed in 96-well reaction plates using a CFX384 Real Time PCR detection system (Bio-Rad^®^, Hercules, CA) with two technical replicates for each of the tested gene. Each 20-μl reaction mixture contained 10.0 μl of 2 × master mix iQ SYBR Green Supermix^®^, 1.0 μl of primers (10μM), 8 μl of PCR-grade H_2_O, and 1 μl of target diluted DNA solution. PCR amplification products were monitored via intercalation of SYBR-Green. The PCR protocol consisted of an initial denaturation step at 95°C for 2 min, 45cycles of amplification, each of which consisted of 15s of denaturation at 95°C, 15s of annealing at 59°C, and 1 min of elongation at 72°C. The quantitation cycle Cq was obtained and exported into an Excel file for further analysis. In order to compare data from different conditions, Cq values were normalized to the Cq value (Kim et al., 2003) of the average of the reference genes actin and unigene 98.

### Gene Differential Expression Identification

Gene expression values were calculated for each library using Fragments Per Kilobase of transcript per Million mapped reads (FPKM). A final clean transcripts count matrix was applied to the statistical package DESeq2 v1.14.1 (Love et al., 2014), which provided negative binomial generalized linear models to test differential expression across treatments, tissues, and clones. Transcripts differential expression was conducted by DESeq2 count matrix input protocol using collapsing technical replicates function and took other factors as background when comparing two levels in one specific factor. The *P*-value for each differentially expressed transcript (DET) was adjusted using the Benjamini and Hochberg’s approach for controlling the false discovery rate (Colquhoun, 2014). The moderated log fold changes proposed by Love et al. (2014) used a normal prior distribution, centered on zero and with a log_2_ scale that has been normalized with respect to library size that is fit to the data. In this study, transcripts with an FDR < 0.05 and | log_2_ fold change| ≥ 1 were considered differentially expressed. PCA analyses were applied to all samples, root samples, and needle samples, respectively, using the R ggplot2 package v3.2.1 (Wickham, 2016).

### Gene Annotation and Network Analysis

Functional annotation of transcripts was performed using the Blast2GO Professional suites v5.2.4 (Gotz et al., 2008). All the transcripts were queried against the NCBI database using NCBI Blast and InterProScan default Blast2GO settings. The results of both searches were merged. Gene Ontology terms were retrieved accordingly when available from the database hits for each transcript. GO enrichment analysis was performed on the annotated sequences to show the abundant and scarce GO terms in upregulated and downregulated DRTs in each clone compared to the reference transcriptome. The Fisher’s exact test and Gene Set Enrichment Analysis (GSEA) were conducted for the enrichment analysis. The significance of the enriched GO terms was plotted by GOplot v1.0.2 (Walter et al., 2015) according to the DRGs in root and the enrichment results from Blast2GO. KEGG pathways map were extracted from the enzyme code assignments in Blast2GO. EnTAP v0.4.7 executed a combined sequence similarity search against three public databases, including: UniProt, TAIR (*Arabidopsis*), and NCBI’s RefSeq Complete protein database (Hart et al., 2020). Gene family assignment was run through the EggNOG database (Huerta-Cepas et al., 2019), and ontology terms were assigned from all taxonomic sources of Gene Ontology. EnTAP was run with default coverage parameters and included a contaminant filter for bacteria, fungal, and insect sequences.

TFs were annotated by searching the PlantTFDB v4.0 (Jin et al., 2017) using the protein sequences of all transcripts obtained with TransDecoder. The Blast2GO and EnTAP annotation results were searched for TF family names from the PlantTFDB classification scheme and for the keywords DNA-binding, DNA binding, TF, regulation of gene expression, and regulation of transcription. The annotation entry of retrieved transcripts encoding TF but with no obvious affiliation to a specific TF family was further inspected to identify gene symbols associated with families, i.e., DREB, which belongs to the ERF family. Gene symbols of matching genes from *Arabidopsis thaliana* were searched on the TAIR database (Berardini et al., 2015). Protein sequences of a few transcripts encoding potential TFs were used to determine homology to known TFs through sequence similarity searches against proteins on the NCBI-BLAST nr database using Blastp with default parameters (Johnson et al., 2008).

Protein sequences of genes deposited in the DroughtDB (Alter et al., 2015) were retrieved from the TAIR10 gene set (Berardini et al., 2015) when present in *A. thaliana* or from DroughtDB itself. Homologous genes to these sequences were searched among the TransDecoder set of ∼60,000 transcripts from this study using a tBlastn local search approach (Camacho et al., 2009). The Blast results were parsed with an in-house perl script. Transcripts with at least 60% sequence identity over more than half the length of drought genes were considered homologous sequences. Transcripts with 50–60% sequence identity with drought genes but with alignments containing 10% or more gaps were also considered homologous sequences. In transcripts with homology with multiple entries in DroughtDB, only the blast hit with the highest sequence percentage identity was retained.

## Results

### Physiological Measurements of Drought Effects in Loblolly Pine Genotypes

**FIGURE 1:**
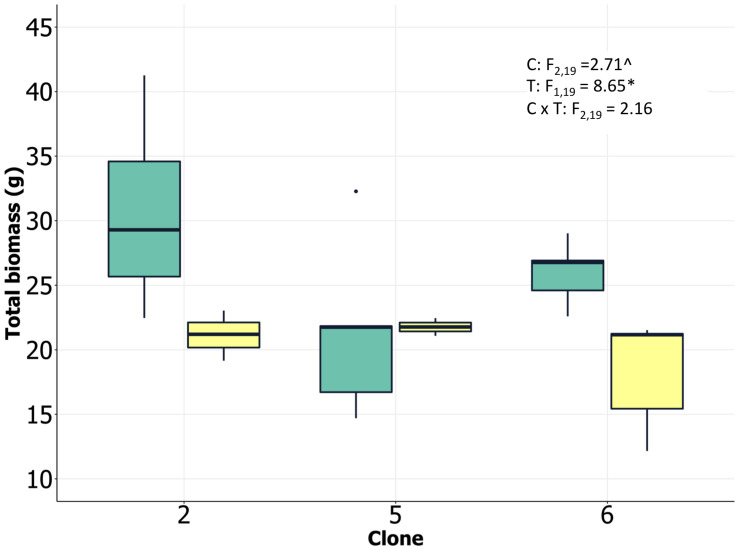
Loblolly pine biomass at harvest (clone 2 and clone 5 selected for transcriptomic analysis) growing under low and high soil water content. Analysis of variance results shown as inset (C, clone; T, treatment; C × T, clone/treatment interaction; *P* < 0.10, ^∗^*P* < 0.05).

We analyzed ramets from multiple loblolly pine clones in randomized experimental greenhouse plots with two water treatments, herein referred to as control (high) and drought (low). Drought treatments successfully reduced soil water potential by approximately 0.5 MPa across all three clones (Supplementary Figure 1). Although clones were statistically significantly different as well, the magnitude of the difference was small and not likely biologically meaningful. Drought significantly reduced total biomass of all clones, although variation among individuals was relatively high and clone 5 appeared to show limited total biomass change, in spite of the lack of significant clone × treatment interaction (Figure 1). There was an indication of differences in plant size among clones, with clone 2 having the largest individuals, and an apparent reduction in allocation to roots by clone 5 in response to the drought treatment. The statistical significance of these patterns was weak, however (*P* < 0.10; Supplementary Figure 2), suggesting caution in interpreting them. Clone 2 exhibited the highest leaf N concentration, consistent with significant investment in photosynthetic enzymes and rapid growth. Lower soil moisture availability reduced specific stem hydraulic conductivity and water potential at 50% loss of conductivity for all clones (Supplementary Figure 2). This reduction in conductivity and xylem vulnerability coincided with increases in C isotope ratios, indicating greater stomatal closure and water use efficiency in response to the drought treatment (Supplementary Figure 2). Integrating all of the available data suggested that there were strong treatment effects on both physiological and growth processes and that the clones had inherent differences in growth and tissue allocation that likely resulted in differential responses to the drought treatment (including numerous processes we were unable to directly measure). We postulate that the physiological and growth results suggest a more high-resource/low tolerance strategy by clone 2 and a greater drought tolerance strategy by clone 5. These two clones were selected for transcriptomic analysis based on these differences.

### Genetic Distance Between the Clones and the Reference Genome

Clones with different genetic distances from the reference genome could lead to biases in the levels of reads mapping and affect transcript abundance quantification. We found no significant difference in the genetic distance between the libraries of the two sequenced clones and the reference genome (*P-*value: 0.61; Supplementary Table 4). Accordingly, the proportion of mapped reads was comparable between the two clones after removing an outlier library in clone 5 with a much higher number of mapped reads (Supplementary Table 2). Moreover, we observed a similar number of transcripts between the two clones for the root tissues compared to needles (Supplementary Table 3).

### Transcriptome Response to Simulated Drought in Loblolly Pine Root

Transcripts that were differentially expressed between drought and control experiments were defined drought-related transcripts or DRTs. Transcripts that were not differentially expressed in our analyses are labeled non-DRTs hereafter. The principal component analysis of transcript data by library showed a tight clustering of control libraries compared to drought library, and a clear separation between libraries of the two clones (Supplementary Figure 4). One of the control root libraries in clone 5 (L1, Supplementary Table 2) clustered more closely to drought libraries of the same clone, suggesting that this ramet was mislabeled. We identified DRTs after removing the L1 library or including it as a library from a drought sample. The analysis with L1 labeled as a drought library produced fewer DRTs, proving more conservative (Supplementary Table 6). We therefore used only DRTs identified including L1 with drought libraries.

**FIGURE 2:**
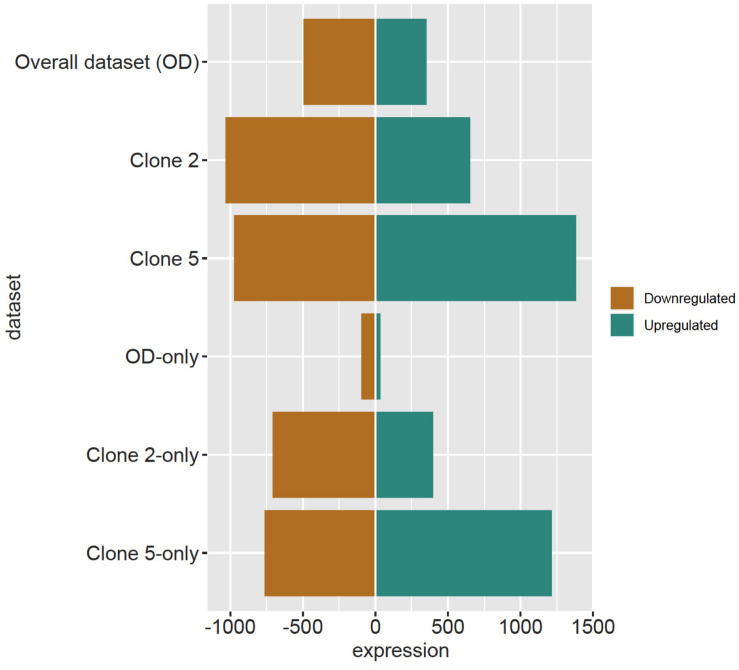
Downregulated and upregulated DRTs found in each dataset. Top three rows: total DRTs. Bottom three rows: dataset-specific DRTs.

The DESeq2 analysis revealed 4012 and 29 DRTs in root and needle tissues, respectively (Table 1 and Supplementary Tables 7, 8). The expression of 12 and 10 DRTs in the root and needle tissues were further analyzed using RT-qPCR. We found a stronger positive correlation (*r*^2^ = 0.69548) between RNA-seq and RT-qPCR results between drought and control in root compared to needle (*r*^2^ = 0.54585) (Supplementary Figure 5). Given the low number of DRTs found in the needles and the lower correlation between RNA-seq and RT-qPCR data, we focused exclusively on the root data in the remainder of the study. We performed separate analyses to detect DRTs in either the overall dataset or in each individual clone. DRTs identified using data from the overall dataset contained more downregulated than upregulated transcripts (Figure 2 and Table 1; “Overall dataset”; Supplementary Figure 6, “RD” and “RU” ellipses). The majority of DRTs in the overall dataset were included in the DRTs separately observed in clone 2, clone 5, or both. For instance, of the 502 downregulated DRTs identified in data from the overall dataset (Supplementary Figure 6, “RD” ellipse), 201, 92, and 103 were also found among upregulated DRTs in clone 2, clone 5, or in both individual clones.

**TABLE 1:** Root up- and downregulated DRTs and non-DRTs.

	DRTs		Non-DRTs	
		
	Upregulated	Downregulated	Upregulated	Downregulated
Clone 2	662	1,041	22,105	21,591
Clone 5	1391	981	23,038	21,563
Overall dataset (OD)	362	502	23,262	22,802
Clones 2 and 5 opposite	3	14	7332	7469
Clone 2-only	405	718	196	195
Cone 5-only	1,223	773	381	281
OD-only	43	106	0	0
Clones 2 and 5-only	2	5	0	0
Clone 2 and OD-only	167	201	4960	5405
Clone 5 and OD-only	67	97	4673	4287
All combined	85	103	13,473	12,859
Total	2,009	2,020	31,803	30,522
				

The differential expression analysis of each clone separately indicated a remarkably higher number of upregulated DRTs in clone 5 compared to clone 2 (Figure 2 and Table 1). Unexpectedly, the two clones also exhibited very little overlap of their DRTs: only 4.4% of upregulated and 5.6% downregulated DRTs were shared between clones 2 and 5 (Table 1 and Supplementary Figure 6). Overall, we identified only 87 upregulated DRTs and 108 downregulated DRTs shared between clones (Supplementary Table 9). Furthermore, a higher number of clone-specific transcripts were found in clone 5, especially upregulated ones, compared to clone 2 (Table 1, “Clone 2-only” and “Clone 5-only”). Seventeen DRTs showed opposite expression patterns between clones, 14 of which were upregulated in clone 5 and downregulated in clone 2 (Table 2). Most of these DRTs encode for enzymes implicated in a variety of functional processes (Table 2). We also identified 802 transcripts with opposite expression pattern between clones that are differentially expressed only in one clone (Supplementary Table 10). The average difference in LFC (log_2_ fold change) between clones for the 819 transcripts with opposite expression patterns was 6.1.

**TABLE 2:** Seventeen DRTs with opposite interclonal expression pattern.

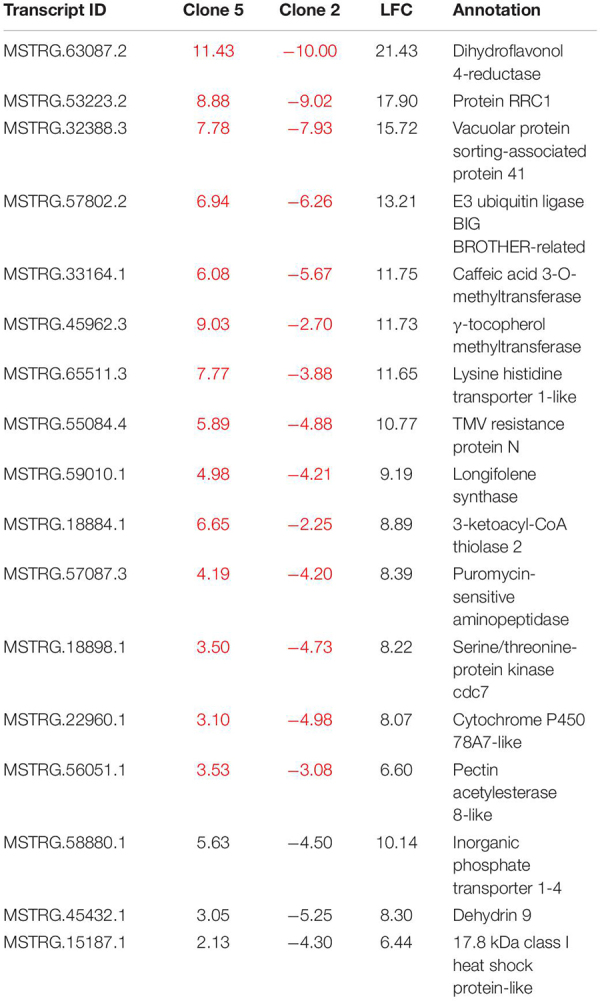

**DRTs upregulated in clone 5 and downregulated in clone 2 are shown in red. LFC: log_2_ fold change.**

To further assess the level of variation between clones, we analyzed the 47,117 transcripts with no significant differential expression between control and drought treatment but with substantial expression levels (mean number of reads per base ≥ 5), or non-DRTs. Although by definition the expression of these transcripts did not change significantly, we labeled non-DRTs as “upregulated” and “downregulated” according to the directionality of their expression change in control vs. drought treatments. We found similar numbers of “up-” and “downregulated” non-DRTs in the overall dataset, and in clones 2 and 5 (Table 1). However, 14,818 non-DRTs showed opposite expression patterns between clones, with 7335 “upregulated” transcripts in clone 2 and 7483 transcripts “upregulated” in clone 5 (Supplementary Tables 11, 12). Of these non-DRTs, 3455 shared at least a twofold opposite LFC between clones. As for DRTs, clone 5 exhibited a higher number of transcripts compared to clone 2 (Table 1). Altogether, these findings underlie the fundamental difference in the gene expression response to soil dehydration between the two genotypes.

**FIGURE 3:**
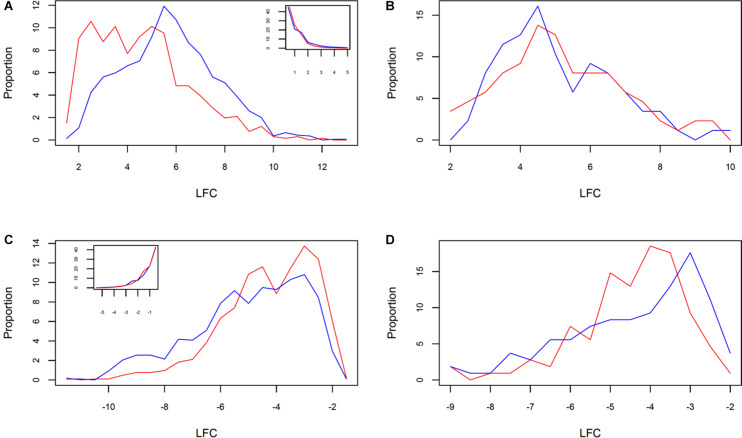
Distribution of LFC in clone 2 (red) and clone 5 (blue) between **(A)** all upregulated and **(B)** downregulated DRTs, and shared **(C)** upregulated and **(D)** downregulated DRTs. The inset in **(A,B)** show the correspondent LFC distributions for non-DRTs.

The analysis of DRTs expression level revealed another facet of the divergent response between the two clones. Both up- and downregulated DRTs in clone 5 showed a significantly higher absolute log_2_ fold change, or (LFC), than the DRTs in the correspondent expression regimes in clone 2 (Supplementary Table 13; upregulated DRTs, Mann–Whitney *U-*test, *P* = 0; downregulated DRTs, Mann–Whitney *U-*test, *P* = 3.55271e-15). The distribution of LFC was higher at lower (LFC) in both clones and expression regimes with the exception of the upregulated DRTs in clone 5, which peaked at around LFC = 5.5 (Figures 3A,B). When the DRTs of both clones were combined, the (LFC) was significantly higher in upregulated compared to downregulated transcripts (Supplementary Table 13; Mann–Whitney *U-*test, *P* = 0). In non-DRTs, (LFC) was also significantly more elevated in “up-” and “downregulated” transcripts of clone 5 than clone 2 (Supplementary Table 13; “r2u-nonDRTs” and “r5u-nonDRTs”, Mann–Whitney *U-*test, *P* = 0; “r2d-nonDRTs” and “r5d-nonDRTs”, Mann–Whitney *U-*test, *P* = 0.013). Given the distribution of the LFC of non-DRTs (insets in Figures 3A,B), the significance of these results is likely the product of a high number of data points rather than reflecting a biologically relevant difference in expression levels between non-DRTs of the two clones. Interestingly, the average [LFC] was not significantly different between the 87 upregulated DRTs and the 108 downregulated DRTs shared by clones (Supplementary Table 13; Wilcoxon Rank test, *P* > 0.05 for both tests). The LFC distribution of the 87 shared upregulated DRTs mirrored that of the upregulated DRTs of clone 5, with a slightly lower central peak around LFC = 4.5 in both clones (Figures 3C,D).

### Functional Annotation of DRTs

**FIGURE 4:**
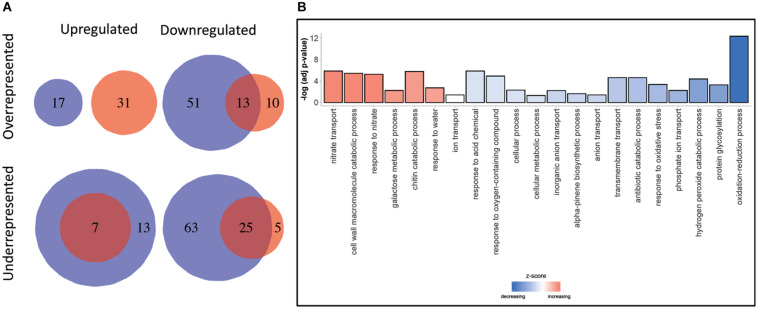
**(A)** GO terms enrichment and depletion between clones and expression regimes. Blue circles: clone 2. Red circles: clone 5. **(B)** Gene ontology terms enrichment for Biological Processes. Decreasing and increasing *z*-scores refer to underrepresented and overrepresented GO terms in DRTs, respectively.

A total of 48,676 and 38,679 transcripts were functionally annotated by Blast2GO and EnTAP, respectively. Of these, 35,838 were annotated by both programs, with a total of 51,538 transcripts showing evidence of functional annotation (Table 3). Using the Fisher’s test implemented in Blast2GO, we found 190 Gene Ontology categories that were significantly enriched or depleted among clones and expression regimes (up- and downregulated DRTs). A larger number of over- and underrepresented GO terms were found in downregulated DRTs when compared to upregulated DRTs (Figure 4 and Supplementary Table 14). Depleted GO categories were largely shared across both clones, whereas the few enriched categories that overlapped between clones 2 and 5 were found only among downregulated genes (Figure 4). Enriched GO terms included categories that are expected in drought response experiments, such as “response to water” and “response to abiotic stimulus” in upregulated DRTs in clone 2 and “response to stimulus” in upregulated DRTs in clone 5 (Supplementary Table 14).

**TABLE 3:** Summary of transcript functional annotation results.

	Blast2GO	EnTAP	Shared	Total
Sequence Homology	48,699	38,279	35,475	51,503
InterPro	47,386	–	–	47,386
GO	37,938	20,916	17,757	41,097
OrthoGroups	–	38,066	–	38,066
Total	48,699	38,679	35,840	51,538

**FIGURE 5:**
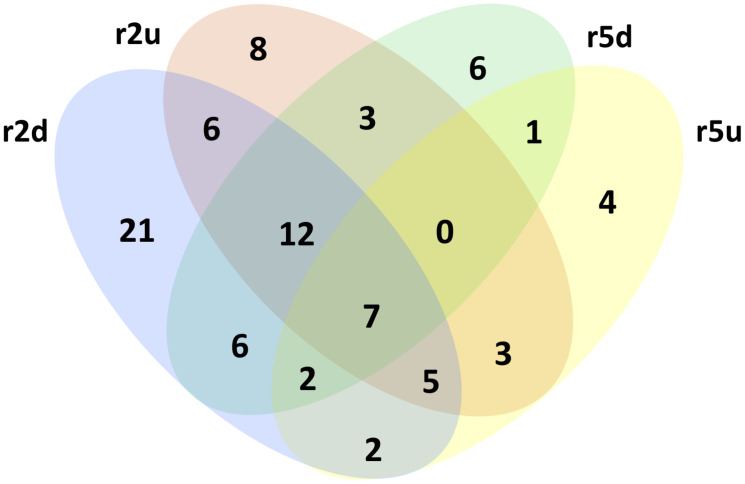
KEGG pathways in upregulated and downregulated genes of clone 2 and clone 5.

Eighty-seven KEGG pathways were found to be associated to 293 up- and downregulated DRTs from the two clones (Table 4). Overall, a higher number of KEGG pathways were found in clone 2 than in clone 5, and in downregulated compared to upregulated DRTs. About 45% of KEGG pathways (39/87) were present only in one clone and one expression regime, but shared pathways were found between both clones and expression regimes, with 7 pathways present in all four types of DRTs (Figure 5). The number of KEGG pathways showed a weak correlation (*r* = 0.38) with the total number of DRTs in each tested clone by condition. Indeed, only 24 KEGG pathways were represented in the group of 1391 upregulated DRTs in clone 5, as opposed to the 44 pathways associated to the 662 upregulated DRTs in clone 2 (Table 4). This suggests that most DRTs in clone 5, and especially those upregulated in response to drought, are largely not associated with metabolic pathways.

**TABLE 4:** Number of KEGG pathways, enzymes, and DRTs in KEGG pathways for up- and downregulated DRTs in clone 2, clone 5, and between the two clones.

	#Pathways	#Enzymes	#DRTs in Pathways
r2d	61	46	106
r5d	37	29	46
r2u	44	34	58
r5u	24	23	63
RD	18	15	12
RU	20	18	20

In 24 KEGG pathways, DRTs encoded enzymes involved in multiple reactions and thus more likely to represent important metabolic components of the drought response in loblolly pine (Supplementary Table 15). For instance, in clone 2, five reactions were affected by downregulated DRTs in the starch and sucrose metabolism pathway (map00500; Supplementary Figure 7A). Overall, several of these 24 pathways included DRTs across both clones or expression regimes (Supplementary Figures 7A–D and Supplementary Table 16). However, only 28/109 enzymatic reactions and a mere 6/293 DRTs were shared between clones and expression regimes across all KEGG pathways, indicating that different components of the same pathways are often activated in the two clones in response to drought (Supplementary Tables 16, 17). Overall, we found a few pathways with multiple enzymatic reactions that showed upregulated or downregulated DRTs only. The pathways “Pyruvate metabolism,” “Pentose and glucuronate interconversions,” and “Thiamine metabolism” contained downregulated DRTs of both clones, whereas several upregulated DRTs in clones 2 and 5 belonged to “Glutathione metabolism,” “Amino sugar and nucleotide sugar metabolism,” and “Galactose metabolism” pathways (Supplementary Table 15). These metabolic reactions could belong to a core group of pathways activated or repressed in response to drought in loblolly pine.

### DRTs That Encode TFs

**FIGURE 6:**
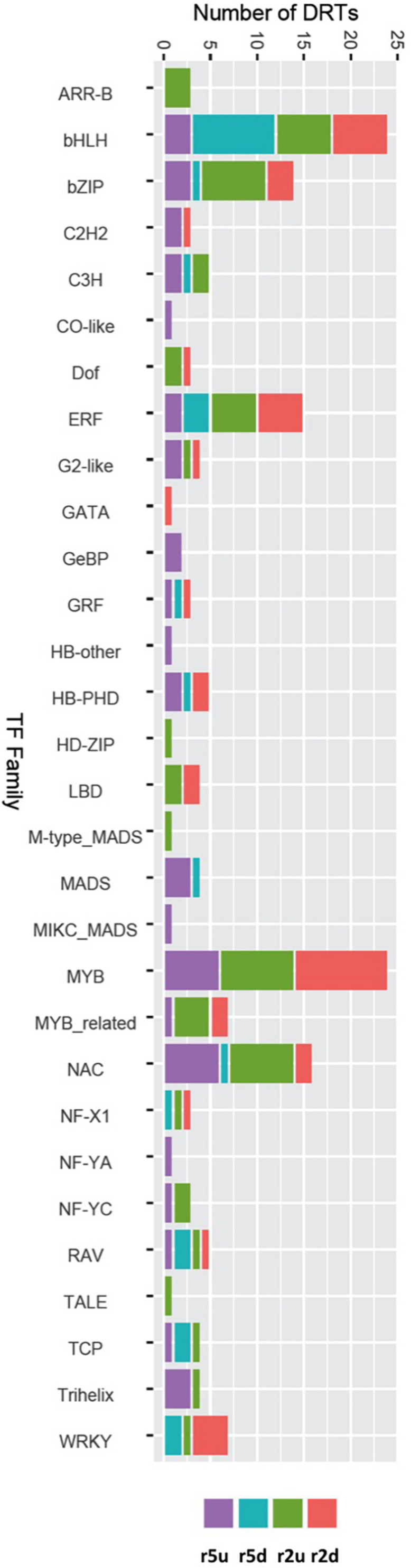
DRTs annotated as transcription factor in each expression regime and grouped by TF families.

To gain further insights into the gene regulatory processes associated with drought tolerance in loblolly pine, we searched for transcripts predicted to encode TFs. A total of 1984 and 1574 transcripts were predicted to encode TFs according to the Blast2GO and EnTAP annotation results, respectively. We also identified 2110 transcripts with homology to known plant TF genes using the PlantTFDB (Jin et al., 2017). Combining these results on a gene-by-gene basis to remove redundancy due to alternative transcripts, we obtained 1,550 predicted loblolly pine TFs encoding genes, corresponding to ∼4.4% of the 35,220 loblolly pine genes of our transcriptome. All TFs were assigned to families according to the PlantTFDB classification. A total of 153 DRTs encoded TFs, with 15 TF-DRTs shared between clones (Figure 6, Tables 5, 6, and Supplementary Table 18). A higher proportion of TFs was found in upregulated DRTs (3.6–9.5%) compared to downregulated DRTs (2.8–4.4%) and in clone 2 compared to clone 5 (Table 5). Additionally, more TF families were identified among upregulated than downregulated DRTs (29 vs. 19). Upregulated DRTs from clone 2 showed the highest proportion of TFs, which was driven by a higher-than-average number of transcripts in multiple families rather than more TF families being present only in this clone and expression regime (Table 5).

**TABLE 5:** Number of predicted TFs in all transcripts, both clones and both regimes.

	# Genes	TF	% TFs	TF families
All transcripts	35,220	1,550	4.4	56
Non-DRTs	31,858	1,397	4.4	56
DRTs	3362	153	4.6	30
r2u	598	57 (11)	9.5	20
r5u	1240	45 (11)	3.6	21
r2d	972	43 (4)	4.4	16
r5d	896	25 (4)	2.8	12

**Numbers in parenthesis show shared DRTs between up- or downregulated regimes.**

**TABLE 6:** Number of predicted TFs in all transcripts, both clones and both regimes.

TF family	All genes	Non-DRTs	r2u	r5u	r2d	r5d	%DRTs
ARR-B	14	11	3	0	0	0	21.4
bHLH	158	136	6 (1)	3 (1)	6 (1)	9 (1)	15.2
bZIP	56	44	7 (2)	3 (2)	3	1	25.0
C2H2	72	68	0	2	1	0	4.2
C3H	44	40	2 (1)	2 (1)	0	1	11.4
CO-like	11	10	0	1	0	0	9.1
Dof	18	15	2	0	1	0	16.7
ERF	164	149	5	2	5	3	9.1
G2-like	27	25	1 (2)	2 (2)	1	0	14.8
GATA	21	20	0	0	1	0	4.8
GeBP	10	8	0	2	0	0	20.0
GRF	8	6	0	1	1 (1)	1 (1)	37.5
HB-other	10	9	0	1	0	0	10.0
HB-PHD	14	9	0	2	2	1	35.7
HD-ZIP	36	35	1	0	0	0	2.8
LBD	47	43	2	0	2	0	8.5
M-type_MADS	12	11	1	0	0	0	8.3
MADS	60	56	0	3	0	1	6.7
MIKC_MADS	8	7	0	1	0	0	12.5
MYB	165	143	8 (3)	6 (3)	10	0	14.5
MYB_related	75	69	4	1	2	0	9.3
NAC	77	62	7 (1)	6 (1)	2	1	20.8
NF-X1	18	15	1	0	1	1	16.7
NF-YA	7	6	0	1	0	0	14.3
NF-YC	11	9	2 (1)	1 (1)	0	0	27.3
RAV	18	15	1	1	1 (1)	2 (1)	27.8
TALE	12	11	1	0	0	0	8.3
TCP	29	26	1	1	0	2	13.8
Trihelix	61	57	1	3	0	0	6.6
WRKY	64	59	1	0	4 (1)	2 (1)	10.9

**Numbers in parenthesis show shared DRTs between up- or downregulated regimes.**

Overall, DRTs encoded TFs from 30/56 families found in the loblolly pine transcriptome (Table 6). Several TF families showed a biased distribution among clones and expression regimes (Table 6). Of the 30 TF families found in DRTs, only five (bHLH, bZIP, ERF, NAC, and RAV) occurred among all clones/regimes, whereas two (NF-YC and Trihelix) were present only in upregulated DRTs of both clones, two (Dof and LBD) were found exclusively in clone 2 DRTs, and one (MADS) occurred only in clone 5 DRTs. A higher proportion of TFs in the NAC and C3H families was found in upregulated DRTs from both clones, whereas the family WRKY contained mostly downregulated genes. Furthermore, 17 and 10 TF families occurred in upregulated or downregulated DRTs of only one clone, respectively.

### DRT Occurrence in a Database of Manually Curated Drought-Related Genes

To determine whether loblolly pine DRTs include orthologs to genes known to be involved in drought tolerance in flowering plants, we searched for sequence homology between DRTs and the 200 genes deposited in DroughtDB, a manually curated database of loci whose role in drought tolerance has been experimentally determined (Alter et al., 2015). We found significant sequence similarity (see section “Materials and Methods”) between 160 loblolly pine transcripts from 116 loci and 83 DroughtDB genes (Table 7 and Supplementary Table 19). The higher number of loblolly pine transcripts than DroughtDB genes is due to both the presence of multiple expressed isoforms in some loblolly pine genes and the duplication of some DroughtDB genes in loblolly pine (Supplementary Table 19). Eleven DRTs matched DroughtDB genes. Seven of these DRTs are predicted to be involved in ABA biosynthesis, catabolism, or downstream pathways (Supplementary Table 19). Nine out of 11 DRTs were upregulated, a significantly higher proportion than downregulated genes (Table 7; Fisher’s exact test, *P* = 0.035). Furthermore, the nine upregulated DRTs exhibited a significantly higher increase in gene expression than all upregulated DRTs combined (Table 7; Mann–Whitney *U-*test, *P* = 0.0015). Two of these DRTs, MSTRG.33848.1, and MSTRG.57622.1, showed conserved expression patterns in clones 2 and 5 (Supplementary Table 19). MSTRG.33848.1 is predicted to encode a beta-carotene hydroxylase involved in the ABA biosynthesis pathway, whereas MSTRG.57622.1 represents a putative pyrroline-5-carboxylate synthase involved in the synthesis of proline. The 149 non-DRTs with homology to DroughtDB genes occurred in both clones with the exception of two transcripts detected only in clone 5. No significant [LFC] differences were found between up- and downregulated transcripts of the two clones. However, 45 of these transcripts had opposite expression patterns between clone 2 and clone 5 (Table 7).

**TABLE 7:** Loblolly pine transcript homology with DroughtDB genes.

	DRTs		Non-DRTs	
		
	Upregulated	Downregulated	Upregulated	Downregulated
Clone 2	6	1	65	75
Clone 5	5	1	73	75
Overall dataset (OD)	6	0	72	75
Clones 2 and 5 opposite	0	0	21	24
Clone 2-only	2	1	0	0
Clone 5-only	1	1	0	2
OD-only	0	0	0	0
Clones 2 and 5-only	0	0	0	0
Clone 2 and OD-only	2	0	12	14
Clone 5 and OD-only	2	0	15	11
All combined	2	0	43	49
Total	9	2	97	100

## Discussion

The genetic basis of drought response variation among different populations is poorly understood in conifers. In this study, we performed a transcriptome analysis on root samples of loblolly pine ramets from two clones with different tolerance to water deficit. This represents one of the most extensive expression study in loblolly pine seedlings grown in drought-simulated conditions and provides a more comprehensive gene expression profile compared to previous array-based studies. We found that the vast majority of drought-related transcripts, or DRTs, exhibit a GxE pattern of expression between the two clones. For instance, although the direction of expression change was largely the same among all genes between clones, twice as many upregulated genes under drought stress were found in clone 5, the more drought-tolerant clone. This suggests that increased drought tolerance in some loblolly pine genotypes may be associated with the ability to activate a larger group of genes compared to drought-sensitive genotypes. Approximately 20% of DRTs (819/4012) showed an opposite expression pattern between clones, including many transcripts with significant differential expression only in one clone. Furthermore, both up- and downregulated DRTs in clone 5 showed a significantly higher absolute log_2_ fold change compared to those of clone 2. Extensive GxE effects were also observed in the 47,117 non-DRTs, with 14,818 transcripts showing opposite expression patterns between clones and 1053 transcripts present only in clone 2 or clone 5. A caveat to this conclusion is that the reduced expression changes observed in non-DRTs might be more sensitive to differences among replicates.

The overall level of GxE interactions was less pronounced at the level of predicted gene functional categories or metabolic pathways. The gene ontology and metabolic pathways enrichment analyses indicate that similar functional groups of transcripts are differentially expressed under water stress in clone 2 and clone 5. However, upregulated DRTs showed no shared biological processes between the two clones. Altogether, these findings lend support to the notion that water deficiency elicits a response based on remarkably different genes and genetic networks at the root level in the two loblolly pine genotypes examined here. This conclusion is further supported by the analysis of differentially expressed TFs, which showed that only 11/102 upregulated TFs and 4/66 downregulated TFs were shared between the two clones.

These results are in contrast with a previous microarray-based analysis pointing to a strong similarity in gene expression patterns between drought-stressed, well-watered, and drought-recovered treatments in roots across four loblolly pine clones (Lorenz et al., 2011). The use of different genotypes, treatment regimes, and gene expression detection approaches is likely responsible for the variation in GxE prevalence between the two studies. Notably, low levels of GxE have also been reported in the root transcriptome of different genotypes exposed to drought stress among flowering plant species. The wheat-tolerant cultivar JM-262 and the susceptible cultivar LM-2 showed largely overlapping sets of both up- and downregulated DRTs in response to low water availability (Hu et al., 2018). In a different study, four wheat varieties exhibited on average a 51% overlap between root DRTs (Mia et al., 2020). High levels of congruence between DRTs of drought-tolerant and -sensitive genotypes/cultivars have also been reported in rice (Baldoni et al., 2016; Lou et al., 2017), barley (Janiak et al., 2018), maize (Zhang et al., 2019), and poplar (Cohen et al., 2010). Although genotypes with varying drought tolerance clearly show remarkable differences in the gene expression response during water deficiency, these differences appear to be especially pronounced between loblolly pine clones 2 and 5.

We recognize that our conclusions might have been affected by some caveats. Both significantly enriched or depleted functional categories and metabolic pathways contained a relatively small proportion of DRTs, likely a consequence of the limited functional gene annotation in gymnosperms compared to angiosperms. Thus, DRTs in the two loblolly pine clones could be more functionally conserved than suggested by our results. Furthermore, we applied a prolonged drought treatment that mimics more closely the water deficiency regimes experienced by loblolly pine forests (Domec et al., 2015), which might elicit a different genetic response compared to analogous experiments that were aimed at testing more “acute” drought conditions typically enforced for a short period of time. Our results underscore the importance of including prolonged drought stress conditions in studies that aim at understanding the genetic basis of the response to water deficit in conifers.

Transcripts with opposite expression pattern between the two clones are particularly intriguing as potential drivers of drought tolerance and should be considered top candidates for further functional experiments in loblolly pine and other conifers. Many DRTs that were upregulated in clone 5 and downregulated in clone 2 are known to be expressed in response to drought or in drought-tolerant varieties in angiosperms (Table 2). Nine of these DRTs encode for enzymes that are implicated in plant responses to abiotic stress. For instance, the most DET between clones (MSTRG.63087.2, LFC = 21.426) encodes a dihydroflavonol 4-reductase enzyme, which promotes the synthesis of flavonoids (Nakabayashi et al., 2014) in response to abiotic stress, including drought (Wang et al., 2013). A second transcript, MSTRG.18884.1, encodes the enzyme γ-tocopherol methyltransferase responsible for converting γ-tocopherol into α-tocopherol, a Vitamin E family member involved in plant stress tolerance and the response to lower soil moisture availability (Munné-Bosch, 2005; Ma et al., 2020). The large superfamily of cytochrome P450 (CYP450) is implicated in a variety of biosynthesis and detoxification pathways associated with plant growth and response to abiotic stress (Jun et al., 2015). Overexpression of cytochrome P450 78A7 led to increased drought tolerance in transgenic rice (Nam et al., 2014). Volatile organic compounds (VOCs) are also known to be involved in plant responses to abiotic stress (Possell and Loreto, 2013). Longifolene synthase participates in the anabolism of VOCs and has been reported to be upregulated in drought-stressed Masson pines (Quan and Ding, 2017). Multiple proteases, such as the puromycin-sensitive aminopeptidase expressed in rice roots and in loblolly pine clone 5 (Wang et al., 2011), are upregulated under water deficit conditions to mobilize nitrogen (Kohli et al., 2012). Pectin acetylesterase 8 removes acetyl-moieties from pectins and contributes to remodeling the cell wall (Wu et al., 2018), which has been associated to the maintenance of cell turgor in the presence of water deficit in angiosperms and in gymnosperms (De Diego et al., 2013; Le Gall et al., 2015). Caffeic acid O-methyltransferase (COMT) is an essential enzyme in the biosynthetic pathway of the lignin precursors monolignols. Lignin synthesis has been shown to increase under low soil moisture conditions in a variety of flowering plants (Moura-Sobczak et al., 2011; Li et al., 2016). The upregulation of circadian rhythm proteins, such as serine/threonine-protein kinase cdc7, might be linked to the decreased mitotic activity observed in water-stressed root tissues of maize (Sacks et al., 1997) and other angiosperms (Kitsios and Doonan, 2011). The peroxisomal 3-ketoacyl-CoA thiolase 2 (MSTRG.18884.1) is a critical component of the ABA signal transduction (Jiang et al., 2011). Finally, E3 ubiquitin ligase BIG BROTHER plays a role in cell proliferation in the root of *A. thaliana* (Cattaneo and Hardtke, 2017). Other notable genes with opposite interclonal expression patterns include dehydrin 9, a member of a family associated with low water availability in seed plants (Hanin et al., 2011), and a lysine histidine transporter with a role in shuttling the ethylene precursor 1-aminocyclopropane-1-carboxylic acid (Choi et al., 2019).

Clone-specific genetic networks involved in abiotic stress responses can be activated or repressed by modified expression of key TFs. Therefore, we prioritized the identification of differences in TFs expression between clones and treatments. Transcripts encoding for TFs from a variety of families were identified among DRTs. Many of these TFs are known to be expressed in response to drought, including the dehydration response element binding factors (DREBs) of the ethylene responsive factor (ERF) family (Xie et al., 2019), the ABA response elements (ABREs) of the basic leucine zipper (bZIP) domain family (Golldack et al., 2014), and TFs from the WRKY (Tripathi et al., 2014), NAC (Nuruzzaman et al., 2013), and MYB (Baldoni et al., 2015) families. Similar cohorts of TF families were identified in drought-response gene expression experiments in conifers, including loblolly pine (Lorenz et al., 2011), as well as in flowering plants (Janiak et al., 2016). We further identified several TF families that have been increasingly recognized in association with drought and may play a major role in the response to water deficit in loblolly pine. Trihelix TFs, which include the GT factors, are present among upregulated DRTs but do not appear among downregulated DRTs. In angiosperms, some Trihelix TFs are known to be expressed in response to abiotic stress, including drought (Xie et al., 2009; Wang et al., 2016; Yu et al., 2018; Magwanga et al., 2019). The largest group of TFs in our dataset is represented by the basic helix-loop-helix (bHLH) family, which includes several up- and downregulated DRTs from both clones. This family alone is suggestive of the complexity of the regulatory networks involved in the response to drought and similar abiotic stressors in loblolly pine; among the 23 DRTs encoding a bHLH TF, only 1 downregulated DRTs was shared between clones. In agreement with previous studies in conifers, we found that most TFs whose expression changed significantly in response to drought were upregulated. Nevertheless, we observed an elevated number of downregulated TFs in our experiments compared to the microarray results of Lorenz et al. (2011), even though these authors found more downregulated than upregulated DRTs. This implies that the downregulation of TFs may play a more important role than previously recognized in the drought response of loblolly pine.

Among the 200 experimentally characterized drought-related genes reported in DroughtDB, we identified 83 genes with high homology with one or multiple loblolly pine transcripts. Given the relatively stringent thresholds we applied to detect homology, it is likely that more known DroughtDB genes are present in loblolly pine. Additionally, some DroughtDB genes might not be expressed in root tissues. The finding that upregulated DRTs with homology to DroughtDB genes are expressed at higher levels than other upregulated DRTs suggests that this small group of genes might play a critical role in drought response. This is further supported by the fact that six of these genes are involved in ABA biosynthesis, catabolism, or downstream pathways. The role of other DroughtDB genes expressed in loblolly pine in response to aridity is less clear, especially those showing opposite expression patterns between clones. This indicates that while some genes might share a key function in drought response in both angiosperms and conifers, many components of the genetic networks activated and repressed in low water availability conditions could differ between flowering plants and gymnosperms.

## Data Availability Statement

The raw RNA-sequencing data generated for this study can be available through the NCBI Sequence Read Archive, project PRJNA681619. The transcript sequences generated and analyzed for this study have been deposited on the NCBI Transcriptome Shotgun Assembly Sequence Database at DDBJ/EMBL/GenBank under the accession GIYS00000000. The version described in this paper is the first version, GIYS01000000.

## Author Contributions

JL performed transcriptomic data analyses and contributed to manuscript analysis. JBW contributed to the study design, plant treatment, samples processing, physiological data analyses, and manuscript writing. AH contributed to transcriptomic data analyses. JLW contributed to transcriptomic data analyses and manuscript writing. MS contributed to plant treatment. J-CD contributed to physiological data analyses and manuscript writing. CL contributed to the study design, samples processing, and manuscript writing. CC contributed to the study design, samples processing, transcriptomic data analyses, and manuscript writing. All authors contributed to the article and approved the submitted version.

## Conflict of Interest

The authors declare that the research was conducted in the absence of any commercial or financial relationships that could be construed as a potential conflict of interest.

## Footnotes

1 https://sibs.tamu.edu

2 https://github.com/TransDecoder
