# Supplementary material for: Extensive Variation in Drought-Induced Gene Expression Changes Between Loblolly Pine Genotypes
Source: Front Genet. 2021 May 31;12:661440. doi: 10.3389/fgene.2021.661440 (PMC8203665; doi:10.3389/fgene.2021.661440)
Supplement: Supplementary file 2 [file Data_Sheet_2.docx]

**
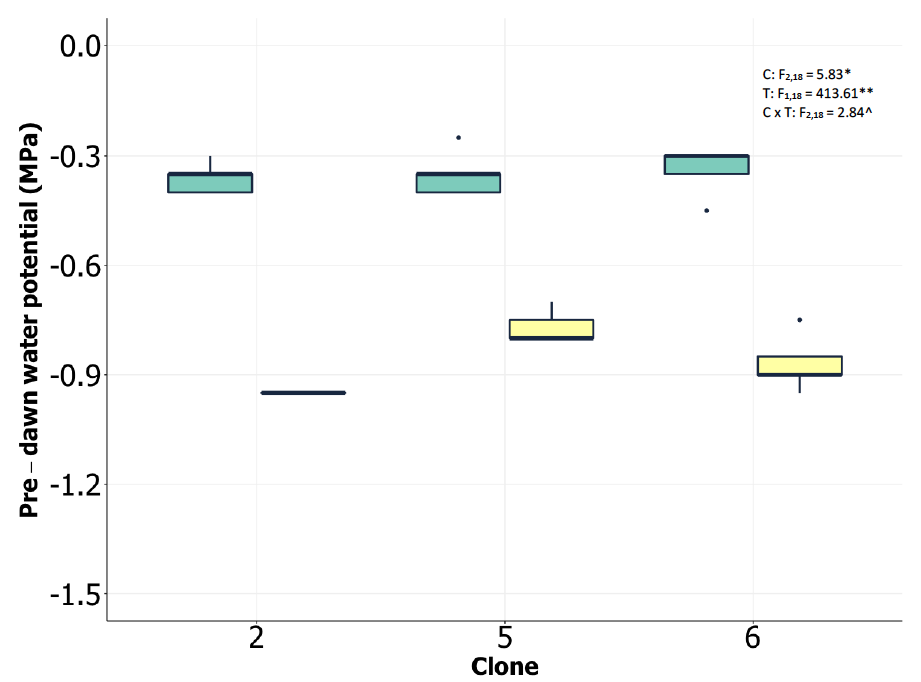
**

**Figure S1**. Pre-dawn water potential of three loblolly genotypes (“2,” “5,” and “6”) at harvest.

Analysis of variance results shown as inset (C = clone, T = treatment, C x T = clone/treatment

interaction; ^P<0.10, *P<0.05, **P<0.01).

**(C)**

**(A)**

**(B)**

| **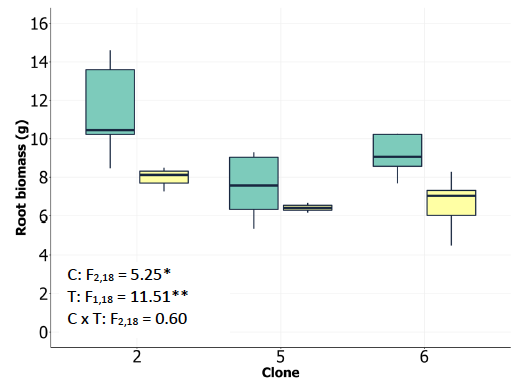**  **(D)** | **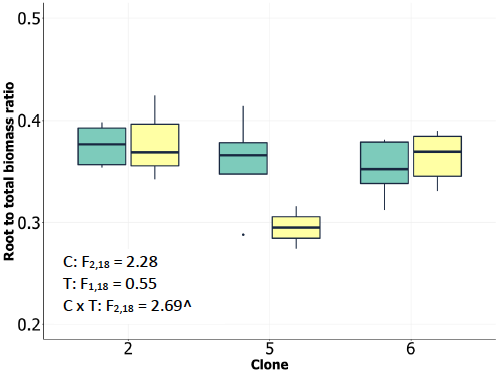** |
| --- | --- |
| **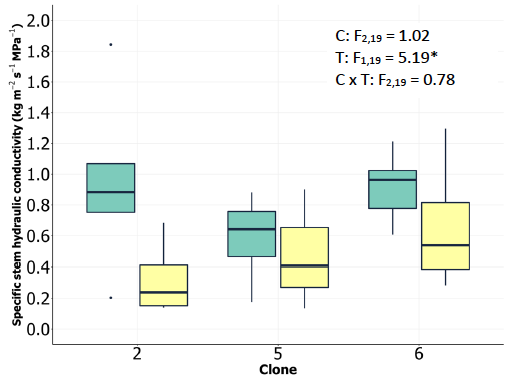**  **(E)** | **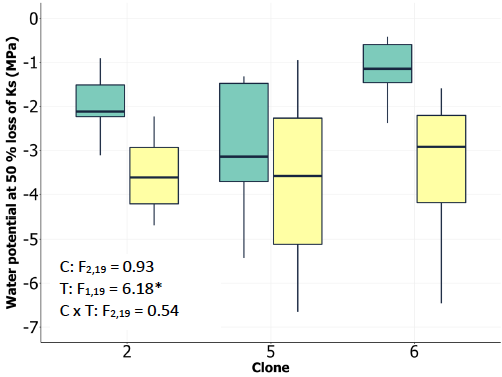**  **(F)** |
|  |  |
| **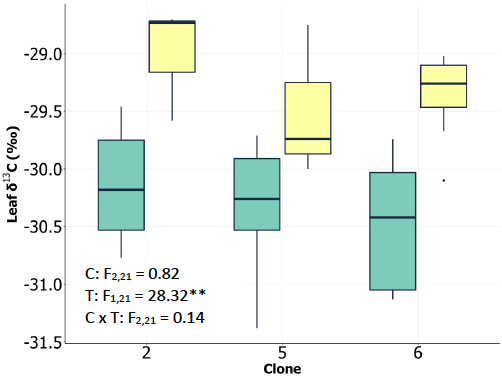** | **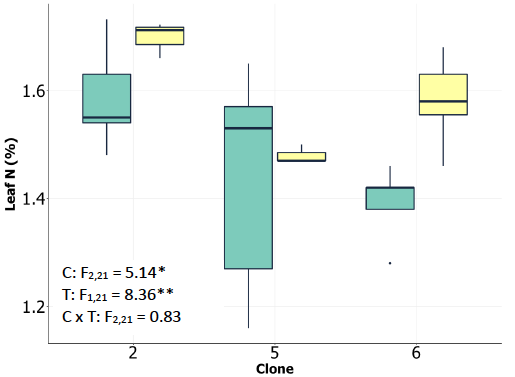** |

**Figure S2**. Physiological measurements of three loblolly genotypes (“2,” “5,” and “6”) grown

under control (shown first in blue) and drought-simulated treatments (shown second in yellow).

(A) Root biomass (g). (B) Root to total biomass ratio. (C) Stem specific hydraulic conductivity

(KS). (D) Stem water potential at which 50% loss of KS occurred (P50). (E) Leaf carbon isotope

ratio (d13C). (F) Leaf nitrogen concentration. In (C) and (D) KS and P50 represent stem water

transport hydraulic efficiency and stem hydraulic safety against drought, respectively. Analysis

of variance results shown as insets for each variable (C = clone, T = treatment, C x T = clone x

treatment interaction; ^P<0.10, *P<0.05, **P<0.01).

**
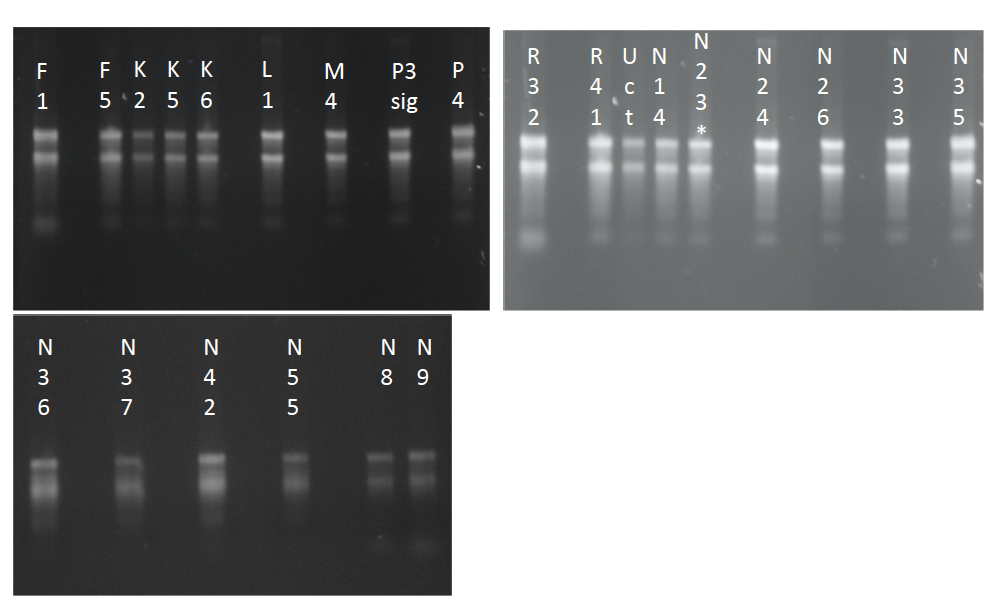
**

**Figure S3**. Agarose gel runs for RNA extracted from the 24 seedlings used to build the RNA-seq libraries. The asterisk labels the sample N23 which was discarded due to contamination.


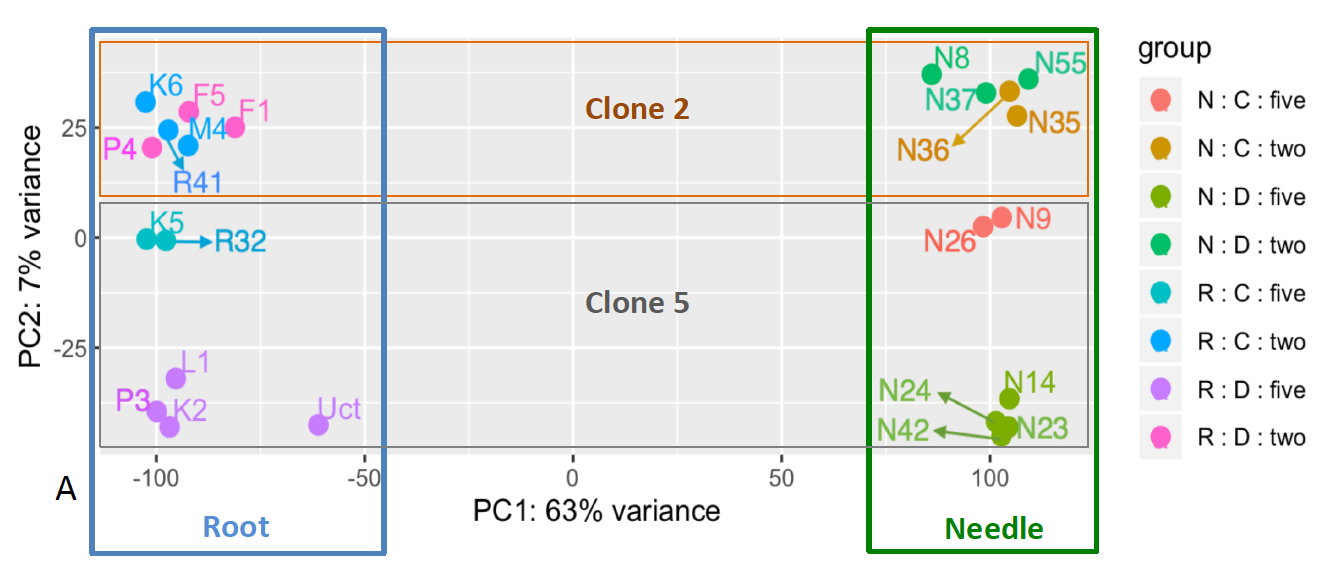

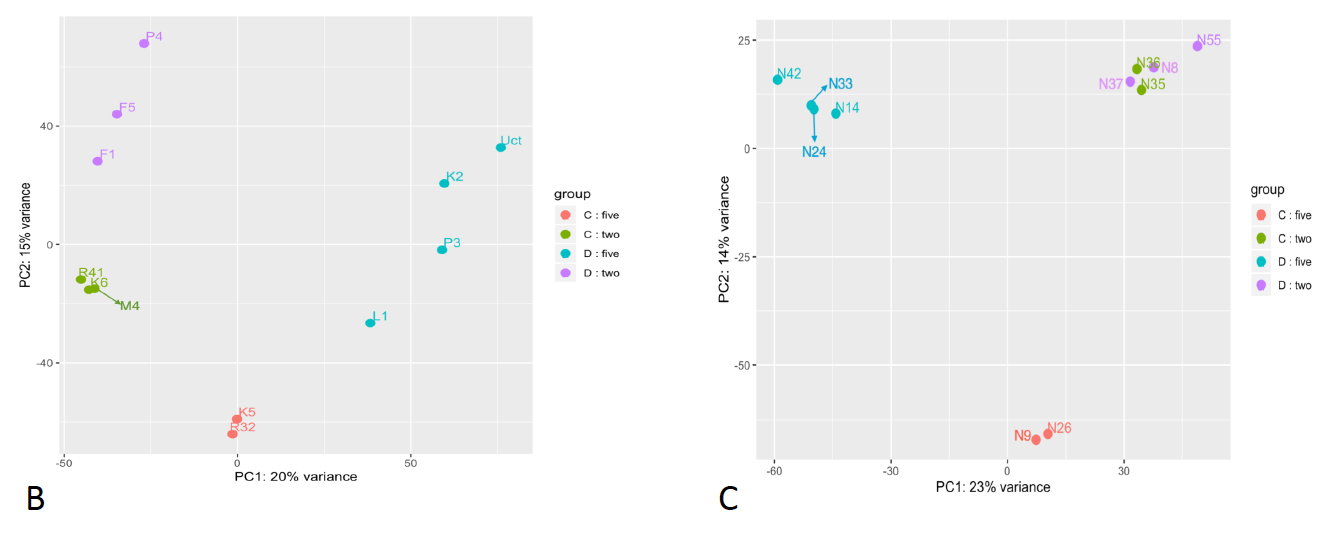


**Figure S4**. Principal component analysis of root and needle libraries. (A) All libraries combined. (B) Root libraries. (C) Needle libraries.

**(A)**

**(B)**

**Figure S5**. Correlation between RNA-seq and RT-qPCR results in thirteen root genes (A) and six needle genes (B).


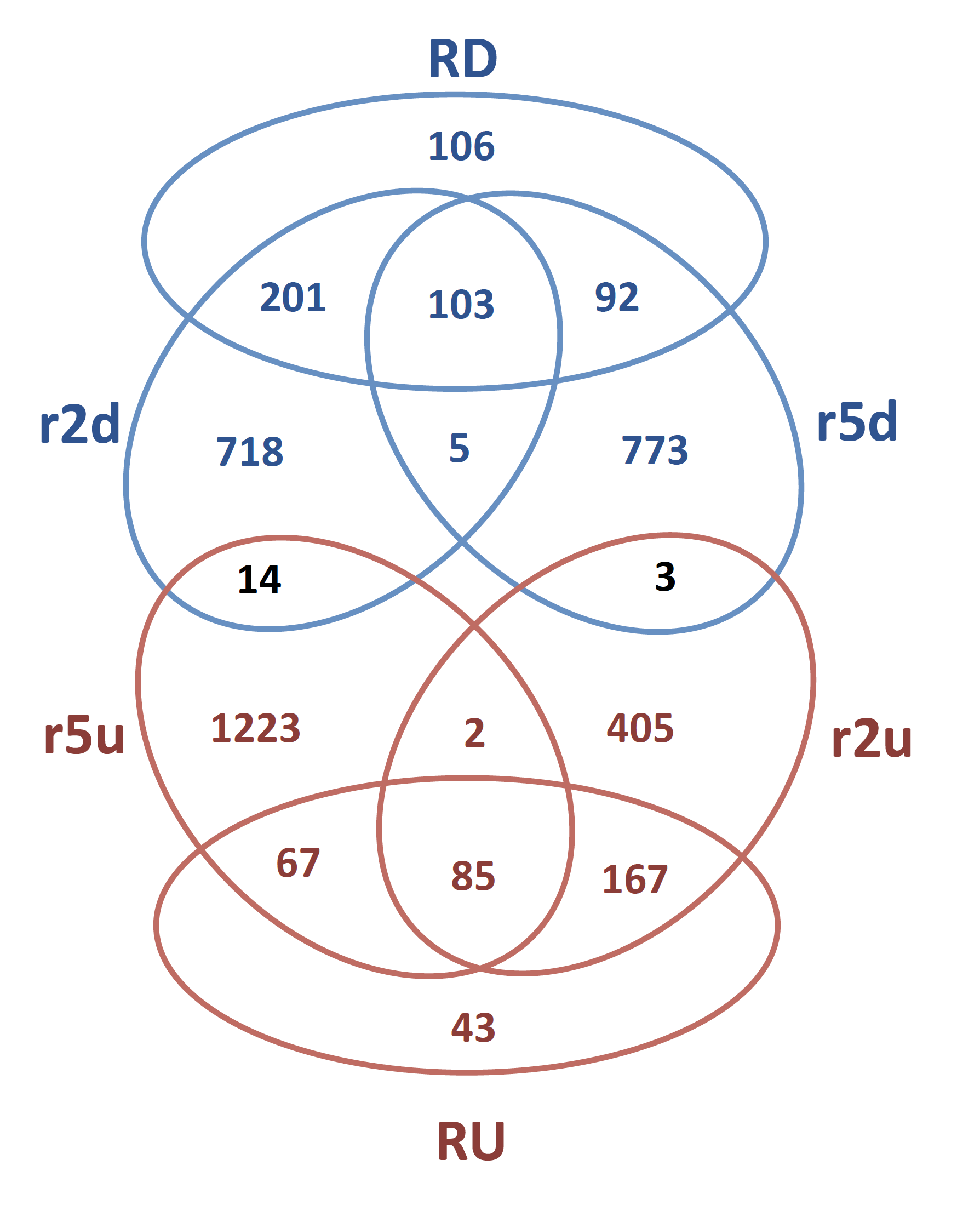


**Figure S6**. Overlap of root differentially expressed transcripts in clone 2 and clone 5. RU: Root Upregulated, overall dataset. RD: Root Downregulated, overall dataset. r2u: root clone 2 upregulated. r2d: root clone 2 downregulated. r5u: root clone 5 upregulated. r5d: root clone 5 downregulated.

**(A)**
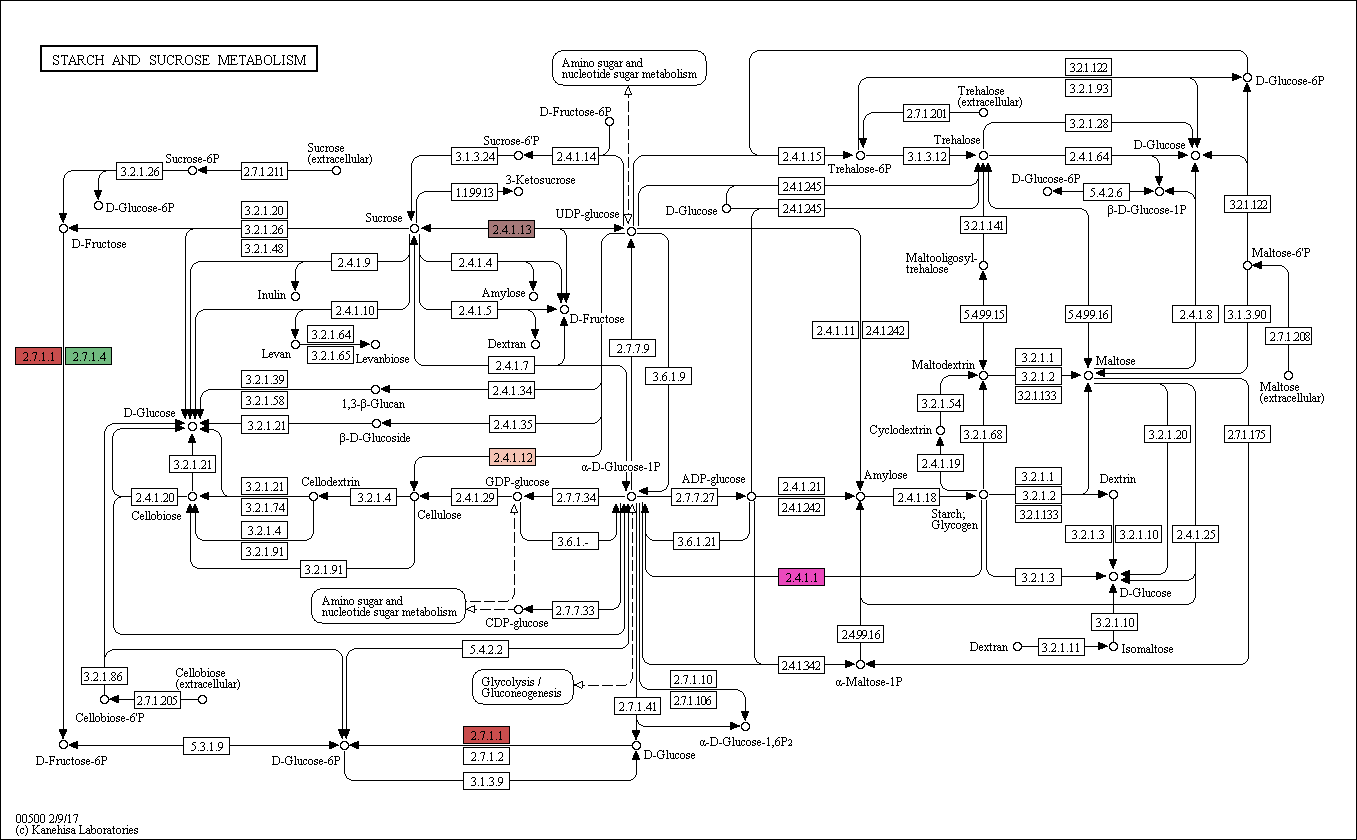


**(B)**


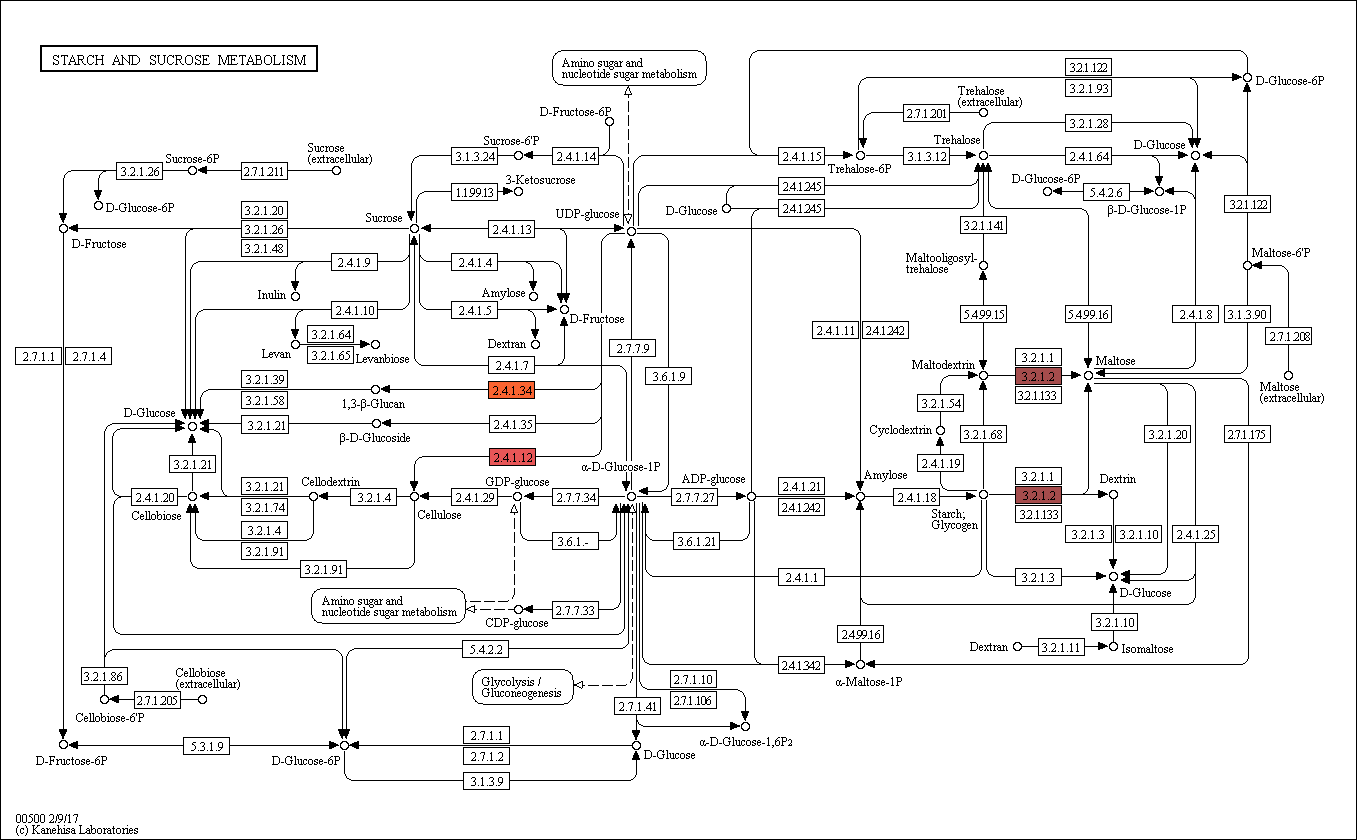


**(C)**

**
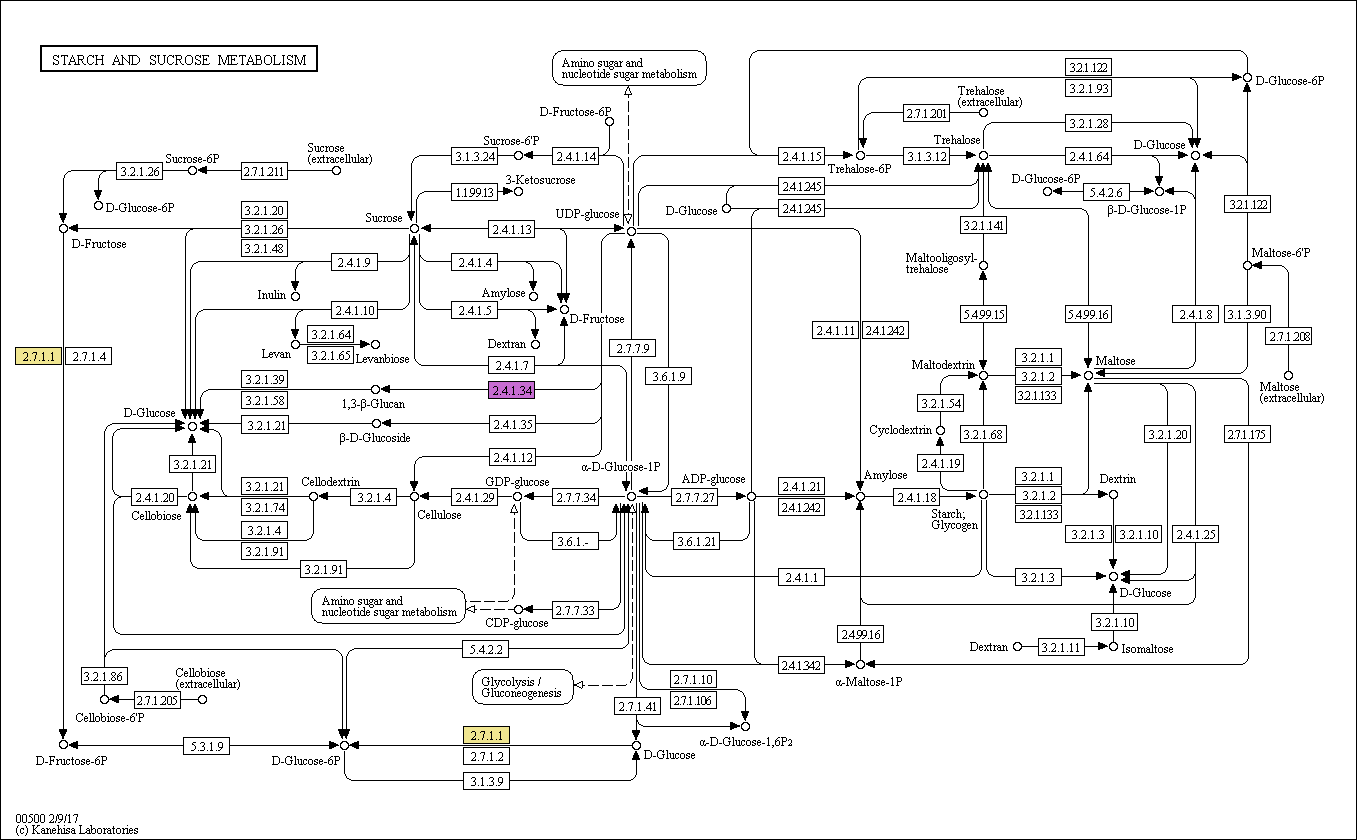
**

**(D)**

**
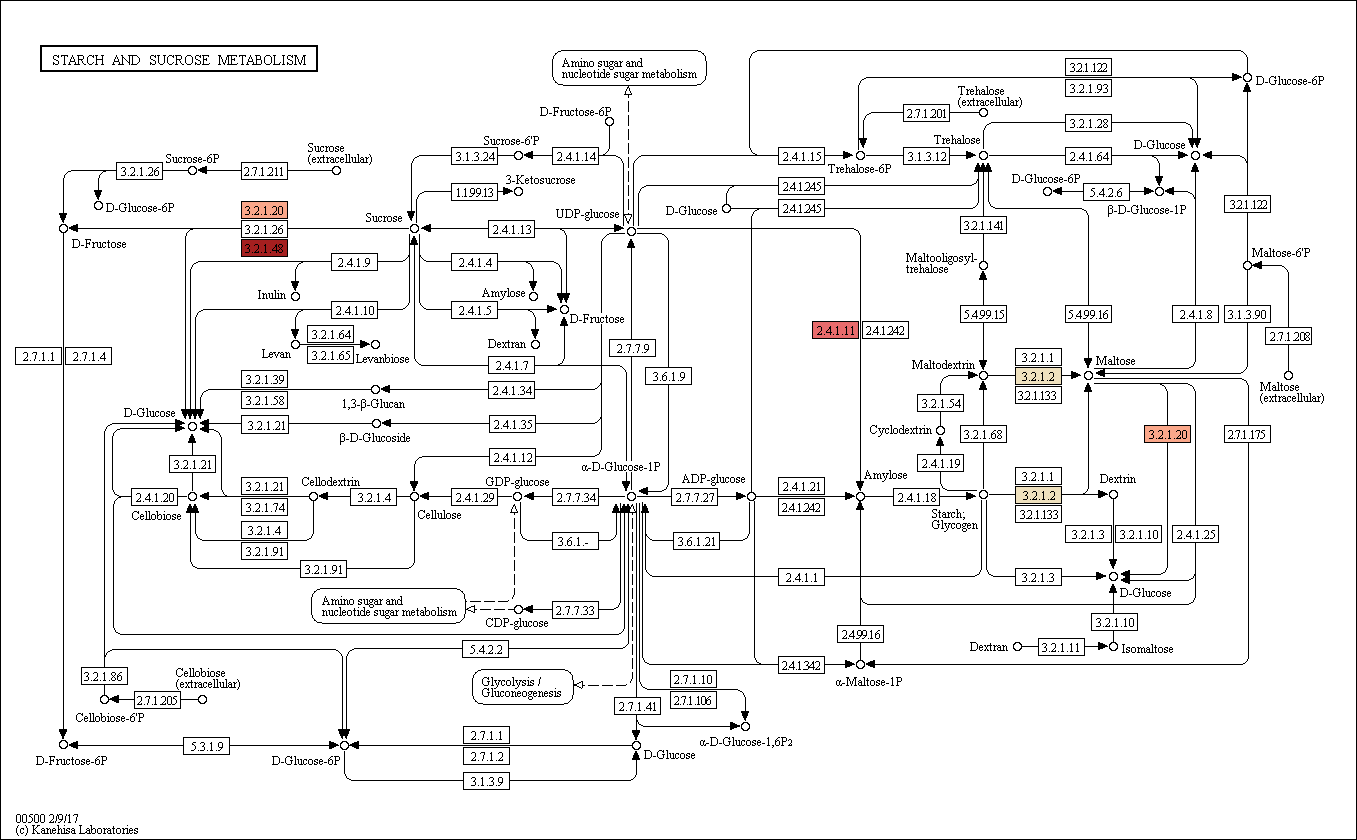
**

**Figure S7**. The KEGG starch and sucrose metabolism pathway. Enzymatic reactions affected by drought are shown by colored boxes showing the Enzyme Commission number.

(A) Downregulated DRTs in clone 2; (B) Downregulated DRTs in clone 5; (C) Upregulated DRTs in clone 2; (D) Upregulated DRTs in clone 5.
